# Early Body Mass Index Trajectory as a Marker of Metabolic and Nutritional Changes in Critically Ill Patients

**DOI:** 10.3390/nu18091396

**Published:** 2026-04-29

**Authors:** Ah Young Leem, Shihwan Chang, Chanho Lee, Mindong Sung, Hye Young Hong, Geun In Lee, Youngmok Park, Seung Hyun Yong, Ala Woo, Sang Hoon Lee, Song Yee Kim, Kyung Soo Chung, Eun Young Kim, Ji Ye Jung, Young Ae Kang, Moo Suk Park, Young Sam Kim, Su Hwan Lee

**Affiliations:** Division of Pulmonary and Critical Care Medicine, Department of Internal Medicine, Severance Hospital, Yonsei University College of Medicine, Seoul 03722, Republic of Korea; yimayoung@yuhs.ac (A.Y.L.);

**Keywords:** body mass index, critical illness, nutritional status, metabolic response, ventilator weaning

## Abstract

**Background:** Body mass index (BMI) is a common nutritional marker, but admission-only measurements present limitations. Early dynamic BMI changes may better reflect metabolic stress and fluid balance. However, the clinical significance of early BMI trajectory during critical illness remains poorly understood. This study evaluated the impact of early BMI trajectory on mortality and ventilator weaning in critically ill patients. **Methods:** This retrospective cohort study included 1355 adult patients (ICU stay ≥ 7 days) admitted to the medical ICU between 2019 and 2025. BMI trajectory was defined as the percentage change from admission to day 7 and was categorized into three groups: decrease (>5% reduction), stable (±5%), and increase (>5% gain). Multivariable Cox proportional hazard and logistic regression analyses were performed to evaluate the association between BMI trajectory and clinical outcomes. **Results:** Of the 1355 patients, 15.9%, 57.7%, and 26.4% were in the decrease, stable, and increase groups, respectively. The increase group demonstrated significantly higher hospital mortality (52.5%) than the decrease (41.9%) and stable (40.0%) groups (*p* = 0.001). Multivariable analysis revealed that an increasing BMI trajectory was independently associated with higher hospital mortality (HR 1.25, 95% CI 1.05–1.48). A decreasing BMI trajectory strongly predicted successful ventilator weaning (OR 2.76, 95% CI 1.81–4.21). **Conclusions:** Early BMI trajectory significantly predicted ICU outcomes. Increasing and decreasing BMI were associated with higher mortality and improved ventilator weaning, respectively. These findings suggest that BMI trajectory may be a simple surrogate marker of metabolic stress, nutritional status, and fluid balance during early critical illness.

## 1. Introduction

Obesity has become a major global health issue and is increasingly prevalent among critically ill patients admitted to the intensive care unit (ICU) [1]. Body mass index (BMI) is widely used to evaluate nutritional status and has been associated with clinical outcomes in critically ill populations [2]. Interestingly, several studies have reported the so-called “obesity paradox”, in which overweight or obese patients demonstrate lower short-term mortality than patients with normal or low BMI during critical illness [3,4]. This paradoxical relationship has been observed in various ICU populations, including patients with sepsis, acute respiratory failure, and cardiovascular disease [5,6].

However, most previous studies have evaluated BMI as a static indicator obtained on ICU admission [2,3]. In the ICU environment, a static, single-point measurement often fails to capture the rapidly evolving metabolic and physiological shifts that are characteristic of critical illness. In critically ill patients, nutritional and metabolic status can change rapidly during the early phase of illness, leading to substantial fluctuations in body weight and BMI within a short duration. These early dynamic changes in BMI, defined as the “BMI trajectory”, could be a more sensitive surrogate for the complex interplay between metabolic stress, systemic inflammation, and fluid homeostasis [7,8]. Therefore, evaluating the dynamic trajectory of BMI during ICU stay may provide additional insights beyond a single baseline measurement regarding metabolic alterations, nutritional changes, and fluid balance during the early phase of critical illness. Furthermore, early weight fluctuations in critically ill patients may represent a combination of catabolic stress, inflammatory responses, and fluid redistribution, all of which are closely linked to clinical outcomes.

Recent research has increasingly focused on trajectory-based analyses of physiological variables in critical illness, such as lactate, Sequential Organ Failure Assessment (SOFA) score, and inflammatory marker trajectories [9,10]. These dynamic approaches have been shown to better capture the evolving pathophysiological state of critically ill patients [10]. Several prior investigations have examined the association between BMI changes and clinical outcomes in critically ill patients. Irving et al. demonstrated the prognostic significance of BMI z-score changes in children with severe sepsis [11], while Zhang et al. reported an association between BMI change and mortality in adult ICU patients [12]. More recently, Liu et al. examined BMI change and short-term mortality in sepsis patients using the MIMIC-IV database [13], while Yang et al. reported the impact of in-hospital BMI variation on 28-day mortality in critically ill surgical patients across multiple centers [14]. Despite the importance of nutritional and metabolic changes during critical illness, the clinical implications of BMI trajectory as a surrogate marker of nutritional and metabolic status remain insufficiently investigated.

In addition to mortality, ventilator weaning success is an important clinical outcome in critically ill patients requiring prolonged mechanical ventilation [15]. Successful weaning is influenced by multiple factors, including respiratory muscle strength, systemic inflammation, nutritional status, and overall disease severity [15,16]. Malnutrition, muscle wasting, and metabolic imbalance are well-recognized contributors to respiratory muscle weakness and delayed ventilator liberation [8,17,18]. Specifically, alterations in BMI during the acute phase can reflect fluctuations in metabolic and nutritional status, which in turn significantly influence respiratory mechanics and weaning outcomes [17,19]. Furthermore, early increase in BMI is often closely linked to positive fluid balance, which exacerbates pulmonary compliance and prolongs mechanical ventilation [20]. Understanding early changes in BMI may provide clinically relevant information regarding the metabolic response to critical illness, nutritional adequacy, and fluid management during ICU stay.

Therefore, this study aimed to investigate the clinical impact of BMI trajectories during the first week of ICU admission in patients admitted to a medical ICU. Specifically, we examined the association between early BMI trajectory and mortality outcomes, including ICU, 28-day, 90-day, and in-hospital mortality. We also evaluated whether BMI trajectory was associated with ventilator weaning success in critically ill patients.

## 2. Methods

### 2.1. Study Population

This retrospective cohort study investigated the clinical impact of BMI trajectories on mortality and weaning success during the first week of ICU admission. The study was conducted at a tertiary referral hospital in South Korea and included all adult patients admitted to the medical ICU (MICU) between 17 May 2019, and 8 December 2025. Initially, 1886 patients admitted to the MICU during the study period were evaluated. To accurately assess the BMI trajectory over the first week, we excluded patients who stayed in the ICU for <7 days (*n* = 531). This minimum 7-day ICU stay criterion was required to allow for meaningful calculation of BMI trajectory, as at least one week of observation is necessary to capture clinically significant early weight changes in critically ill patients. Ultimately, 1355 adult patients who stayed in the ICU for ≥7 days were included in the final analysis.

### 2.2. Data Collection and Definitions

Baseline demographics, including age and sex, as well as baseline BMI, were recorded. BMI was calculated as body weight (kg) divided by the square of height (m^2^). Body weight was measured at ICU admission (Day 0) and on ICU Day 7 using a bedside scale or in patients requiring continuous renal replacement therapy, estimated from dialysis machine weight recordings. Height was measured at admission and assumed to be constant throughout the study period, as serial height measurement is not clinically indicated in the ICU setting. BMI trajectory was defined as the dynamic change in BMI during the first week of ICU admission, calculated using the difference between the baseline BMI at admission and BMI measured on ICU Day 7. Accordingly, changes in BMI trajectory reflect changes in body weight standardized for height, and do not involve serial assessment of body surface area. Based on the magnitude of this change, patients were classified into three trajectory groups: decrease (decrease in BMI > 5% from baseline), stable (BMI change within ±5%), and increase (increase in BMI > 5%). Comorbidities such as hypertension, diabetes mellitus, solid tumors, chronic obstructive pulmonary disease (COPD), congestive heart failure, cerebrovascular accident (CVA), and chronic kidney disease (CKD) were assessed. Disease severity and general health status were also evaluated using the SOFA score and the Charlson Comorbidity Index (CCI). Additionally, the use of mechanical ventilation and continuous renal replacement therapy (CRRT) during ICU stay was documented.

Ventilator weaning failure was defined as death while receiving invasive mechanical ventilation, continuous requirement for invasive mechanical ventilation throughout the entire duration of ICU stay without successful liberation, or conversion to home mechanical ventilation followed by transfer to a general ward or long-term care hospital. Weaning success was defined as successful liberation from invasive mechanical ventilation, achieved either as spontaneous breathing via a tracheostomy airway without any mechanical ventilatory support for 7 consecutive days, or as discharge from the ICU breathing without mechanical ventilatory assistance via a natural or tracheostomy airway, whichever occurred first.

### 2.3. Outcome Measures

The primary outcomes evaluated were in-hospital mortality and weaning success rate. In-hospital mortality was defined as death occurring at any time during the index hospitalization, regardless of ICU discharge status. Post-discharge vital status was ascertained through linkage with the institutional electronic medical record system, which captures all in-hospital deaths comprehensively at this tertiary referral center. All patients were followed until hospital discharge or in-hospital death, whichever occurred first. Secondary outcomes included ICU mortality, 28-day mortality, 90-day mortality, ICU length of stay (LOS), and hospital LOS.

### 2.4. Statistical Analysis

Continuous variables are presented as the mean ± standard deviation (SD) or median with interquartile range (25–75th percentile), depending on the data distribution. Categorical variables are expressed as numbers and percentages. For the comparison of continuous variables among the three groups, one-way analysis of variance (ANOVA) or the Kruskal–Wallis test was used as appropriate. Categorical variables were compared using the chi-squared test or Fisher’s exact test. A multivariable Cox proportional hazards model was utilized to identify independent predictors of hospital mortality, generating hazard ratios (HRs) and 95% confidence intervals (CIs). To evaluate factors associated with weaning success, multivariable logistic regression analysis was performed, reporting odds ratios (ORs) and 95% CIs. For both multivariable models, variables with clinical significance or those showing a trend toward significance (*p* < 0.1) in the univariate analysis were included using a backward stepwise procedure. Survival probabilities and the cumulative incidence of weaning success were estimated using the Kaplan–Meier method, and differences between groups were compared using the log-rank test. All statistical analyses were performed using SPSS software (version 28.0, IBM Corp., Armonk, NY, USA).

## 3. Results

### 3.1. Baseline Characteristics According to the BMI Trajectory Groups

**Figure 1:**
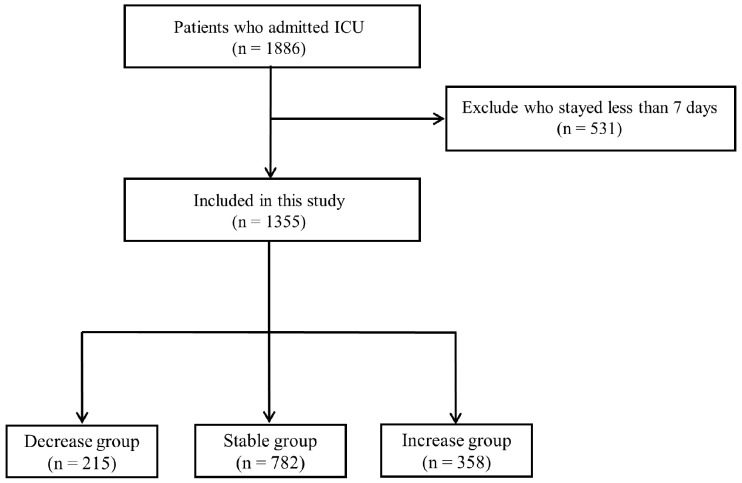
Study flow diagram.

A total of 1355 patients who remained in the intensive care unit (ICU) for ≥7 days were included in the analysis and categorized into three BMI trajectory groups: decrease (*n* = 215), stable (*n* = 782), and increase (*n* = 358) (Figure 1).

The baseline characteristics of the study population according to the BMI trajectory groups are presented in Table 1. The mean age differed significantly among the groups, with patients in the decrease group being younger than those in the stable and increase groups (64.0 ± 14.4 vs. 67.5 ± 13.2 vs. 67.3 ± 14.2 years, *p* = 0.003). The proportion of female patients was higher in the increase group than in the decrease and stable groups (40.5% vs. 30.2% vs. 32.7%; *p* = 0.014). The baseline BMI also differed significantly across the groups (*p* < 0.001). The increase group had the lowest baseline BMI (21.6 ± 4.2 kg/m^2^), whereas the decrease group had the highest baseline BMI (23.7 ± 4.5 kg/m^2^). The prevalence of underweight patients was highest in the increase group (22.9%). Among the comorbidities, CKD was more common in the decrease group than in the other groups (34.0% vs. 24.8% vs. 24.0%, *p* = 0.015). There were no significant differences in hypertension, diabetes mellitus, solid tumors, COPD, congestive heart failure, CVA, or CCI score. Regarding illness severity, the SOFA score differed significantly among the groups (*p* < 0.001). The decrease and increase groups had higher SOFA scores than the stable group. Mechanical ventilation and CRRT use were also significantly more frequent in the increase group (*p* < 0.001 for both).

### 3.2. Clinical Outcomes According to BMI Trajectory

Among the patients discharged alive, ICU LOS showed a trend toward difference among the groups (*p* = 0.062), with the longest stay observed in the increase group (17.5 [11.0–29.2] days), followed by the stable (15.5 [11.0–30.0] days) and decrease (12.0 [9.8–16.2] days) groups (Table 2). Hospital LOS did not significantly differ among survivors (*p* = 0.794). Mortality outcomes varied significantly according to BMI trajectory. The increase group showed the highest ICU mortality (38.3%) compared to that of the decrease (23.7%) and stable (24.7%) groups (*p* < 0.001). Similarly, the increase group had higher 28-day (29.6% vs. 19.5% vs. 19.4%, *p* = 0.001), 90-day (48.3% vs. 36.7% vs. 34.4%, *p* < 0.001), and in-hospital (52.5% vs. 41.9% vs. 40.0%, *p* = 0.001) mortalities.

**Figure 2:**
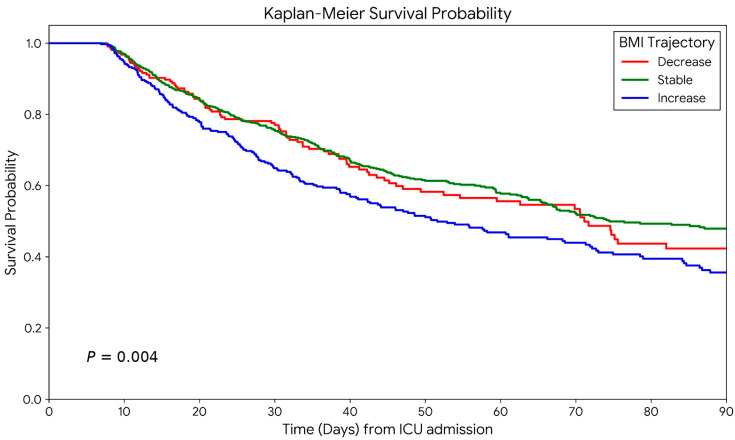
Kaplan–Meier survival probability.

Kaplan–Meier survival analysis also demonstrated a significantly lower survival probability in the increase group than in the other groups (Figure 2).

### 3.3. Multivariable Analysis of Hospital Mortality

The results of the multivariable Cox proportional hazard analysis for hospital mortality are presented in Table 3.

Older age showed a marginal association with in-hospital mortality (HR 1.01, 95% CI 1.00–1.01). A higher CCI score (HR 1.07, 95% CI 1.03–1.10), higher SOFA score (D7) (HR 1.24, 95% CI 1.20–1.28), CRRT use (HR 1.62, 95% CI 1.36–1.93), and mechanical ventilation (HR 1.37, 95% CI 1.07–1.74) were independently associated with increased hospital mortality. Compared with the stable BMI trajectory group, the increase group was independently associated with higher hospital mortality (HR 1.25, 95% CI 1.05–1.48). In contrast, the decrease group was not significantly associated with hospital mortality (HR 1.09, 95% CI 0.87–1.37). In a sensitivity analysis incorporating baseline BMI as an additional covariate, the hazard ratios for both trajectory groups remained virtually unchanged (ΔHR < 0.01), and baseline BMI itself was not an independent predictor of hospital mortality (HR 1.00 per 1 kg/m^2^, 95% CI 0.98–1.02), confirming that the trajectory effect was not confounded by the admission BMI level (Supplementary Table S1).

### 3.4. BMI Trajectory and Ventilator Weaning

The comparison of weaning success rates showed significant differences among BMI trajectory groups. The decrease group demonstrated the highest weaning success rate (76.2%) compared to that by the stable (53.3%) and increase (54.4%) groups (*p* < 0.001). The association between BMI trajectory and ventilator weaning success was evaluated using multivariate logistic regression analysis (Table 4).

**Figure 3:**
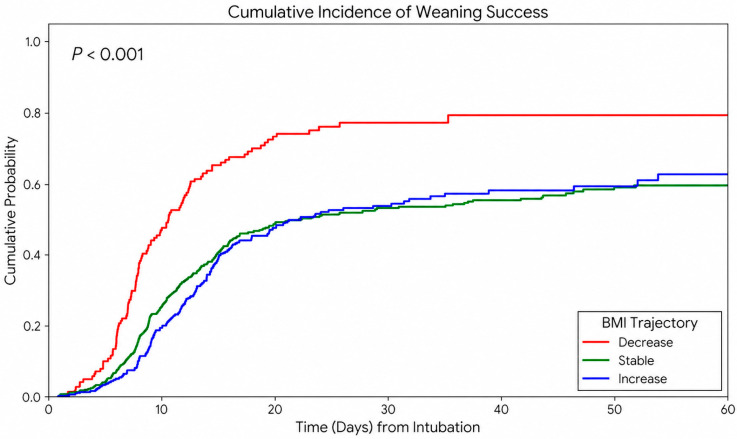
Cumulative probability of weaning success according to the BMI trajectory groups.

A higher SOFA score at D7 was significantly associated with a lower likelihood of successful weaning (OR 0.94, 95% CI 0.91–0.97). Compared with the stable group, the decrease group was significantly associated with higher odds of successful weaning (OR 2.76, 95% CI 1.81–4.21). In contrast, the increase group was not significantly associated with weaning success. Kaplan–Meier analysis of cumulative weaning success also demonstrated significant differences among the BMI trajectory groups (Figure 3).

### 3.5. Model Discriminatory Ability: BMI Trajectory vs. Baseline BMI

To evaluate whether longitudinal BMI trajectory assessment provides superior prognostic discrimination compared to a single baseline BMI measurement, we constructed three multivariable logistic regression models for in-hospital mortality prediction: Model A, incorporating baseline BMI with clinical covariates (age, SOFA score at D7, CCI, CRRT use, and mechanical ventilation) without trajectory information; Model B, incorporating BMI trajectory groups with the same clinical covariates (original manuscript model); and Model C, incorporating both baseline BMI and trajectory groups with all covariates.

All three models demonstrated comparable discriminatory ability (Model A: AUROC = 0.742; Model B: AUROC = 0.743; Model C: AUROC = 0.744). Bootstrap-based pairwise comparisons (2000 iterations) revealed no statistically significant differences between any pair of models (Model B vs. A: ΔAUC = +0.001, *p* = 0.439; Model C vs. B: ΔAUC = +0.001, *p* = 0.623; Model C vs. A: ΔAUC = +0.002, *p* = 0.329). These findings indicate that BMI trajectory and baseline BMI provide statistically equivalent prognostic discrimination, and that their combination does not confer additional discriminatory benefit. ROC curves for all three models are presented in Supplementary Figure S1. To facilitate individualized risk prediction at the bedside, a nomogram was constructed incorporating the variables from the final multivariable Cox model—age, SOFA score at Day 7, CCI score, CRRT use, mechanical ventilation, and BMI trajectory group—for the prediction of 90-day mortality. The nomogram is presented as Supplementary Figure S2.

## 4. Discussion

This retrospective cohort study of critically ill patients evaluated the clinical impact of BMI trajectory during the first week of MICU admission. Our results demonstrated that patients with an increasing BMI trajectory during the first week of ICU stay had significantly higher mortality than those with a stable BMI. In contrast, a decreasing BMI was not associated with increased mortality. Interestingly, a decreasing BMI trajectory was associated with a higher likelihood of successful weaning from the ventilator.

These findings highlight the importance of dynamic metabolic and nutritional changes during early ICU admission rather than relying solely on baseline BMI. Previous studies investigating the relationship between BMI and mortality in critically ill patients have largely focused on admission BMI [2,3]. Several reports have described an inverse association between BMI and mortality, commonly referred to as the “obesity paradox” [3,5]. For example, Pickkers et al. demonstrated that higher BMI was associated with lower hospital mortality in critically ill patients [3]. Similarly, Prescott et al. reported that overweight and obese patients with sepsis had improved survival than normal-weight patients [5].

However, baseline BMI does not adequately reflect the dynamic metabolic and nutritional alterations that occur during critical illness. During the early phase of critical illness, patients often experience profound metabolic stress, characterized by increased catabolism, systemic inflammation, and rapid fluid shifts [7,8]. Unlike prior studies that have characterized the ‘obesity paradox’ using static admission BMI, our findings extend this understanding by demonstrating that the direction of early weight change—rather than the absolute BMI level at admission—carries independent prognostic significance. Specifically, we show that (1) baseline BMI is not an independent predictor of hospital mortality after adjustment for illness severity (HR 1.00, *p* = 0.956), (2) the prognostic association of BMI trajectory is not confounded by admission BMI level (ΔHR < 0.01 after adjustment), and (3) the discriminatory ability of a trajectory-based model is statistically equivalent to that of a baseline BMI model (AUROC 0.743 vs. 0.742, *p* = 0.439). Together, these findings indicate that monitoring the early trajectory of BMI provides clinically equivalent—and potentially more actionable—prognostic information than a single admission measurement while capturing the dynamic interplay between fluid balance, metabolic stress, and nutritional reserve during critical illness. Notably, in the acute phase of critical illness, an increasing BMI trajectory is usually a clinical manifestation of pathological fluid accumulation and ‘fluid creep’ rather than nutritional recovery [21,22]. This phenomenon may be particularly pronounced in patients with a lower baseline BMI, as observed in our increase group (21.6 ± 4.2 kg/m^2^), who may lack sufficient physiological reserve to tolerate excessive volume [23]. The high in-hospital mortality (57.5%) in the increase group suggests that rapid weight gain during the first week reflects a failure of fluid homeostasis compounded by catabolic stress and compromised metabolic reserve, which is particularly detrimental to patients with a lower nutritional reserve on admission—as evidenced by the lowest baseline BMI (21.6 ± 4.2 kg/m^2^) and highest proportion of underweight patients (22.9%) observed in the increase group (Table 1) [23]. We acknowledge that BMI trajectory cannot mechanistically distinguish between fluid-driven weight gain and true nutritional recovery. An increasing BMI during early ICU admission may reflect (1) pathological fluid accumulation from aggressive resuscitation and capillary leak, (2) nutritional repletion, or (3) a combination of both. In our cohort, the cumulative fluid balance differed significantly among trajectory groups (*p* < 0.001), with positive balances observed across all groups: increase group 8.5 [4.8–14.1] L, decrease group 7.2 [−0.3–17.9] L, and stable group 3.4 [1.5–12.3] L. Notably, the divergent BMI trajectories observed despite similarly positive fluid balances in the increase and decrease groups suggest that factors beyond fluid accumulation—including catabolic muscle wasting, nutritional deficit, and disease-related metabolic changes—may also contribute to early BMI trajectory in critically ill patients. Nevertheless, direct body composition measurements—such as bioelectrical impedance analysis or CT-based muscle mass quantification—and granular fluid balance records were not available in this dataset. Future studies incorporating these measures are needed to confirm the mechanistic interpretation and to disentangle the relative contributions of fluid accumulation and nutritional changes to early BMI trajectory.

Our findings suggest that an increasing BMI trajectory during the first week of ICU admission may reflect worsening fluid overload and disease severity. Previous studies have demonstrated that a positive fluid balance is associated with increased mortality in critically ill patients [20]. For instance, Bouchard et al. showed that fluid accumulation is independently associated with mortality in patients receiving renal replacement therapy [24]. Similarly, Rosenberg et al. reported that cumulative fluid overload was independently associated with fewer ventilator-free days and worse clinical outcomes in critically ill patients [25].

Conversely, we observed that patients with a decreasing BMI were more likely to achieve successful ventilator weaning. A reduction in BMI may reflect improved fluid balance or more effective metabolic adaptation during recovery from critical illness. It is noteworthy that the decrease group had the highest baseline BMI (23.7 ± 4.5 kg/m^2^) in our cohort. This suggests that having an adequate metabolic reserve at admission may allow patients to better withstand the stress of critical illness while successfully mobilizing excess fluid during the recovery phase. Fluid overload has been shown to impair lung compliance and gas exchange, which may delay ventilator liberation [25]. Thus, a decrease in body weight and BMI may indicate improved respiratory mechanics and metabolic stabilization, thereby facilitating ventilator weaning.

Another important finding of our study is that the BMI trajectory remained independently associated with hospital mortality after adjusting for major clinical confounders, including SOFA scores at D7. This suggests that early BMI trajectory may serve as a simple and readily available clinical indicator reflecting the combined effects of metabolic stress, nutritional status, and fluid management in critically ill patients. Traditional severity indices, such as SOFA scores, capture organ dysfunction but do not fully reflect the metabolic and nutritional responses to critical illness. Therefore, BMI trajectory may provide complementary prognostic information beyond conventional clinical risk factors.

To overcome this limitation, several tools are available to more precisely differentiate changes in lean body mass, fat mass, and fluid accumulation during critical illness. CT-based muscle mass quantification at the L3 vertebral level provides a validated assessment of skeletal muscle cross-sectional area and has been associated with clinical outcomes in ICU patients. Bioelectrical impedance analysis (BIA) offers a non-invasive bedside method for estimating total body water, fat-free mass, and phase angle—the latter reflecting cellular integrity and nutritional status. Ultrasound-based measurement of rectus femoris cross-sectional area or quadriceps muscle thickness allows for dynamic, real-time assessment of muscle wasting during ICU stay. Daily fluid balance records, when combined with serial body weight measurements, enable the estimation of the fluid-driven component of weight change. Integration of these complementary tools in future prospective studies would substantially advance the mechanistic understanding of BMI trajectory during critical illness and overcome the inherent limitation of BMI as a composite, undifferentiated marker.

This study has several clinical implications. First, monitoring BMI trajectory during the early phase of ICU admission may provide valuable information regarding metabolic and nutritional responses to critical illness. Second, the identification of patients with rapidly increasing BMI, especially those with a low baseline BMI, may help clinicians recognize individuals at a higher risk of adverse outcomes. Third, BMI trajectory may serve as a pragmatic bedside marker integrating nutritional status, metabolic stress, and fluid management during the early course of critical illness.

This study has several limitations. First, this was a retrospective, single-center study, which may limit the generalizability of the findings. Second, BMI trajectory cannot mechanistically distinguish between fluid-driven weight gain, catabolic muscle wasting, nutritional deficit, and disease-related metabolic changes—all of which may simultaneously contribute to early weight changes during critical illness. In our cohort, the observation that divergent BMI trajectories occurred despite similarly positive cumulative fluid balances in the increase and decrease groups (8.5 L vs. 7.2 L, respectively) underscores that fluid balance alone does not fully explain the BMI trajectory, and that multiple mechanistic pathways are likely involved. This fundamental limitation restricts the interpretability of the BMI trajectory as either a nutritional or a fluid management surrogate in isolation. As the BMI trajectory reflects serial weight change standardized for a fixed height, it is mathematically equivalent to a normalized weight trajectory, and future studies directly comparing BMI trajectory, absolute weight trajectory, and cumulative fluid balance as independent predictors of outcomes would further clarify the relative contributions of these measures. Third, BMI trajectory in this study was defined using percentage change, which may be subject to a mathematical floor effect whereby patients with a lower baseline BMI have greater arithmetic capacity for percentage-based increase. Although the correlation between baseline BMI and BMI change was modest (r = −0.181 for percentage change; r = −0.154 for absolute change), and sensitivity analyses confirmed that adjustment for baseline BMI did not materially alter the trajectory-outcome associations (ΔHR < 0.01), future studies should consider incorporating the absolute BMI change as a complementary metric to further validate the robustness of trajectory-based classification. Fourth, the exclusion of patients with an ICU stay of <7 days (*n* = 531) was required to allow meaningful calculation of BMI trajectory; however, this criterion may introduce selection bias. Excluded patients likely represent those with either milder illness (early recovery) or extreme severity (early death), such that our cohort may not fully represent the entire spectrum of critically ill patients. Findings should therefore be interpreted with caution when applied to populations with very short or very long ICU stays. Fifth, CRRT initiation timing varied across trajectory groups (median Day 0 in all groups, but with a wider IQR in the increase group extending to Day 7). As the cumulative I/O balance reported in Table 1 already incorporates fluid removal via CRRT, the net effect of CRRT on BMI trajectory is complex and cannot be fully captured by cumulative balance alone. Nevertheless, the timing and intensity of CRRT prescription may have differentially influenced the fluid dynamics within each trajectory group in ways not fully reflected in the aggregate balance data. Future studies with granular daily fluid balance and CRRT prescription data would better characterize this relationship. Sixth, data on enteral and parenteral nutrition provision during the first week of ICU admission—including timing of initiation, route, volume delivered, and achievement of caloric targets—were not systematically captured in this retrospective dataset. The absence of nutritional therapy data precludes a direct assessment of the contribution of nutrition provision to BMI trajectory and limits the mechanistic interpretation of our findings. Seventh, the total SOFA score at Day 7 was used as a composite measure of organ dysfunction in the multivariable models. The respiratory sub-component of the SOFA score, which directly reflects oxygenation status and may be particularly informative for predicting ventilator weaning outcomes, was not separately analyzed. Future studies examining individual SOFA sub-components as predictors of weaning success may provide additional mechanistic insight. Eighth, ICU LOS and hospital LOS were reported descriptively for survivors only and were not formally adjusted for illness severity in multivariable models, as LOS represents a complex composite endpoint influenced by multiple clinical, logistic, and institutional factors that were beyond the scope of this analysis.

## 5. Conclusions

An increasing BMI trajectory during the first week of ICU admission was independently associated with higher mortality in critically ill patients, whereas a decreasing BMI trajectory was associated with improved ventilator weaning success. These findings suggest that early BMI trajectory may serve as a clinically useful marker reflecting metabolic, nutritional, and fluid status in critically ill patients.

## Figures and Tables

**Table 1:** Baseline characteristics of the study population according to the BMI trajectory groups.

Variable	BMI Trajectory Groups			*p*-Value
	Decrease Group (*N* = 215)	Stable Group (*N* = 782)	Increase Group (*N* = 358)	
Age, years	64.0 ± 14.4	67.5 ± 13.2	67.3 ± 14.2	0.003
Sex				0.014
Male	150 (69.8)	526 (67.3)	213 (59.5)	
Female	65 (30.2)	256 (32.7)	145 (40.5)	
Baseline BMI, kg/m^2^	23.7 ± 4.5	23.3 ± 4.6	21.6 ± 4.2	<0.001
Underweight < 18.5	26 (12.1)	106 (13.6)	82 (22.9)	
Normal 18.5–23	76 (35.3)	292 (37.3)	155 (43.3)	
Overweight 23–25	39 (18.1)	133 (17.0)	64 (17.9)	
Obese ≥ 25	74 (34.4)	251 (32.1)	57 (15.9)	
BMI change, kg/m^2^	−2.2 ± 1.1	−0.0 ± 0.6	+2.3 ± 1.3	<0.001
Cumulative input (D0–D7), L	22.8 [18.7–26.5]	23.9 [20.0–28.3]	25.6 [21.9–29.7]	<0.001
Cumulative output (D0–D7), L	14.9 [1.4–23.8]	18.4 [9.3–23.6]	16.9 [9.5–22.0]	0.003
Cumulative I/O balance (D0–D7), L	7.2 [−0.3–17.9]	3.4 [1.5–12.3]	8.5 [4.8–14.1]	<0.001
Comorbidities				
Hypertension, *n* (%)	139 (64.7)	455 (58.2)	204 (57.0)	0.162
Diabetes Mellitus, *n* (%)	84 (39.1)	305 (39.0)	151 (42.2)	0.577
Solid Tumor, *n* (%)	61 (28.4)	272 (34.8)	133 (37.2)	0.095
COPD, *n* (%)	30 (14.0)	109 (13.9)	43 (12.0)	0.656
Congestive Heart Failure, *n* (%)	24 (11.2)	98 (12.5)	42 (11.7)	0.835
CVA (Stroke), *n* (%)	36 (16.7)	119 (15.2)	72 (20.1)	0.121
CKD, *n* (%)	73 (34.0)	194 (24.8)	86 (24.0)	0.015
CCI Score	3.6 ± 2.3	3.5 ± 2.4	3.5 ± 2.4	0.763
APACHE II Score	27.2 ± 7.9	26.3 ± 8.3	27.8 ± 7.9	0.022
SOFA Score, D0	10.3 ± 4.2	9.4 ± 3.7	10.3 ± 3.6	<0.001
SOFA Score, D7	7.9 ± 4.5	7.5 ± 4.2	8.9 ± 4.3	<0.001
Mechanical Ventilation, *n* (%)	161 (74.9)	673 (86.1)	329 (91.9)	<0.001
CRRT Use, *n* (%)	68 (31.6)	150 (19.2)	91 (25.4)	<0.001
Laboratory findings				
Albumin, g/dL	2.8 [2.6–3.0]	2.8 [2.6–3.1]	2.7 [2.5–3.1]	0.009
Glucose, mg/dL	172.0 [136.0–220.0]	180.0 [141.0–232.0]	180.5 [143.5–243.0]	0.071
WBC, ×10^3^/µL	12.0 [6.2–18.0]	12.2 [8.2–18.2]	12.2 [7.5–19.0]	0.658
Platelet, ×10^3^/µL	116.0 [62.0–199.0]	149.5 [80.0–248.5]	136.0 [69.0–214.5]	<0.001
BUN, mg/dL	35.2 [21.7–56.8]	29.8 [19.2–46.7]	32.6 [23.4–50.7]	0.001
Creatinine, mg/dL	1.5 [0.7–2.6]	1.0 [0.7–1.9]	1.1 [0.7–1.9]	0.002
BUN/Cr ratio	25.6 [17.9–36.6]	26.4 [19.3–37.3]	28.5 [20.1–38.4]	0.048
Total bilirubin, mg/dL	0.8 [0.4–1.9]	0.7 [0.4–1.1]	0.7 [0.4–1.4]	0.004

Note: Continuous variables are presented as the mean ± standard deviation (SD). Categorical variables are presented as numbers (percentage). Abbreviations: BMI, body mass index; COPD, chronic obstructive lung disease; CVA, cerebrovascular accident; CKD, chronic kidney disease; CCI, Charlson Comorbidity Index; APACHE, Acute Physiology and Chronic Health Evaluation; SOFA, Sequential Organ Failure Assessment; CRRT, continuous renal replacement therapy.

**Table 2:** Mortality outcomes according to the BMI trajectory groups.

Outcome	BMI Trajectory Groups			*p*-Value
	Decrease Group (*N* = 215)	Stable Group (*N* = 782)	Increase Group (*N* = 358)	
ICU LOS, days (survivors only)	12.0 [9.8–16.2]	15.5 [11.0–30.0]	17.5 [11.0–29.2]	0.062
Hospital LOS, days (survivors only)	69.0 [37.5–104.5]	61.0 [28.0–104.0]	59.0 [37.0–87.0]	0.794
ICU Mortality	51 (23.7)	193 (24.7)	137 (38.3)	<0.001
28-day Mortality	42 (19.5)	152 (19.4)	106 (29.6)	0.001
90-day Mortality	79 (36.7)	269 (34.4)	173 (48.3)	<0.001
In-Hospital Mortality	90 (41.9)	313 (40.0)	188 (52.5)	0.001

Note: Continuous variables are presented as median (25–75th percentile). Categorical variables are presented as numbers (percentage). Abbreviations: ICU, intensive care unit; LOS, length of stay.

**Table 3:** Multivariable Cox proportional hazards model for hospital mortality.

Variable	Hazard Ratio (95% CI)
Age	1.01 (1.00–1.01)
Male Sex	1.01 (0.86–1.19)
CCI Score	1.07 (1.03–1.10)
SOFA Score, D7	1.24 (1.20–1.28)
CRRT Use	1.62 (1.36–1.93)
Mechanical Ventilation	1.37 (1.07–1.74)
BMI Trajectory (Ref: Stable)	
Increase Group	1.25 (1.05–1.48)
Decrease Group	1.09 (0.87–1.37)

Abbreviations: CCI, Charlson Comorbidity Index; SOFA, Sequential Organ Failure Assessment; CRRT, continuous renal replacement therapy; BMI, body mass index.

**Table 4:** Multivariable logistic regression analysis for weaning success.

Variable	Odds Ratio (95% CI)
Age	1.00 (1.00–1.01)
Male Sex	1.14 (0.88–1.48)
CCI Score	0.88 (0.81–0.94)
SOFA Score, D7	0.94 (0.91–0.97)
CRRT Use	0.84 (0.55–1.28)
BMI Trajectory (Ref: Stable Group)	
Increase Group	1.02 (0.77–1.35)
Decrease Group	2.76 (1.81–4.21)

Abbreviations: CCI, Charlson Comorbidity Index; SOFA, Sequential Organ Failure Assessment; CRRT, continuous renal replacement therapy; BMI, body mass index.

## Data Availability

The complete dataset used in this study will be made available to researchers upon reasonable request from the corresponding author.

## Supplementary Materials

The following supporting information can be downloaded at: https://www.mdpi.com/article/10.3390/nu18091396/s1, Supplementary Table S1: Sensitivity analysis—Cox proportional hazards model for hospital mortality; Supplementary Figure S1: ROC curve overlay comparing Models A, B, and C; Supplementary Figure S2: Nomogram for individualized 90-day mortality prediction incorporating age, SOFA D7, CCI, CRRT use, mechanical ventilation, and BMI trajectory group.

## Abbreviations

The following abbreviations are used in this manuscript:

BMI	Body mass index
CCI	Charlson Comorbidity Index
CKD	Chronic kidney disease
COPD	Chronic obstructive pulmonary disease
CRRT	Continuous renal replacement therapy
CVA	Cerebrovascular accident
HR	Hazard ratio
ICU	Intensive care unit
LOS	Length of stay
MICU	Medical intensive care unit
OR	Odds ratio
