# Supplementary material for: Early Body Mass Index Trajectory as a Marker of Metabolic and Nutritional Changes in Critically Ill Patients
Source: Nutrients. 2026 Apr 29;18(9):1396. doi: 10.3390/nu18091396 (PMC13164838; doi:10.3390/nu18091396)
Supplement: Supplementary file 1 [file nutrients-18-01396-s001.zip › Supplementary Table.pdf]

Supplementary Table S1. Sensitivity Analysis — Cox Proportional Hazards Model for Hospital Mortality

Variable	Original Model (without baseline BMI)	Sensitivity Model (with baseline BMI)	Change in HR
BMI Increase vs. Stable	HR 1.25 (1.05-1.48) P = 0.012	HR 1.25 (1.04–1.48) P = 0.013	ΔHR = 0.00
BMI Decrease vs. Stable	HR 1.09 (0.87–1.37) P = 0.340	HR 1.09 (0.85–1.37) P = 0.341	ΔHR = 0.00
Baseline BMI (per 1 kg/m^2)	—	HR 1.00 (0.98–1.02) *P* = 0.956	—
