# Supplementary figures and images for: Early Body Mass Index Trajectory as a Marker of Metabolic and Nutritional Changes in Critically Ill Patients
Source: Nutrients. 2026 Apr 29;18(9):1396. doi: 10.3390/nu18091396 (PMC13164838; doi:10.3390/nu18091396)

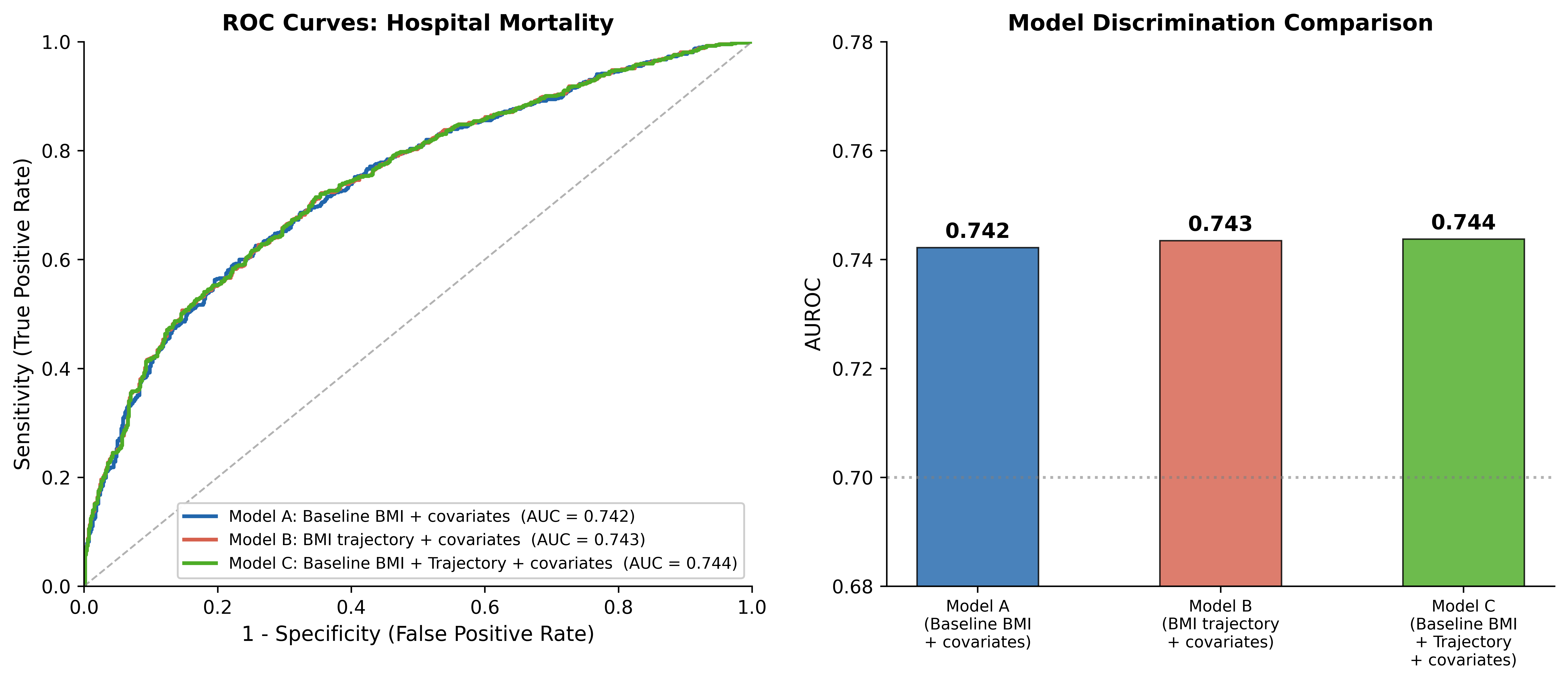

Supplement: Supplementary file 1 [file nutrients-18-01396-s001.zip › Supplementary_Figure_S1_final.tif]

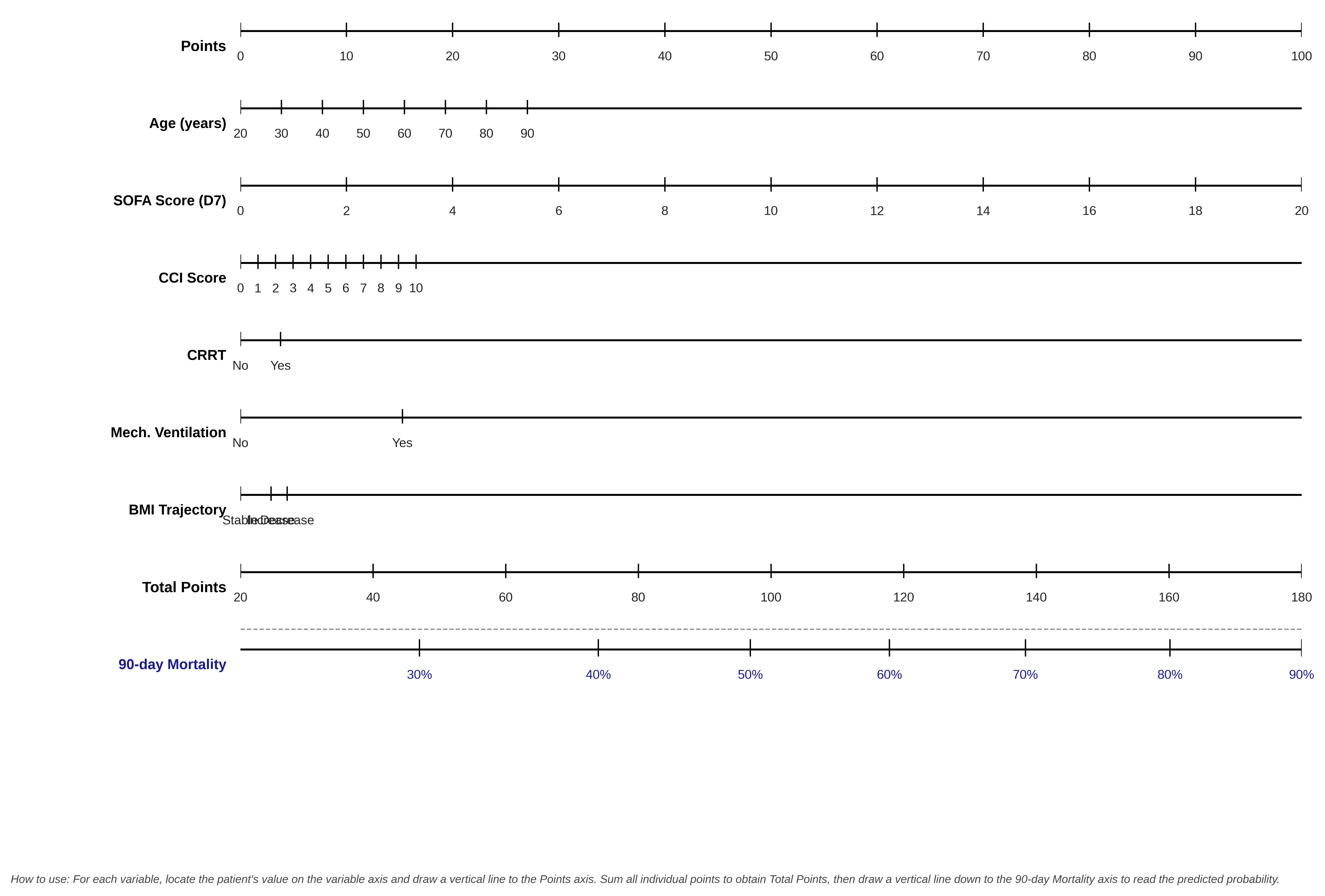

Supplement: Supplementary file 1 [file nutrients-18-01396-s001.zip › Supplementary_Figure_S2_final.tif]
